# Detachment tectonics at Mid-Atlantic Ridge 26°N

**DOI:** 10.1038/s41598-019-47974-z

**Published:** 2019-08-14

**Authors:** Florent Szitkar, Jérôme Dyment, Sven Petersen, Jörg Bialas, Meike Klischies, Sebastian Graber, Dirk Klaeschen, Isobel Yeo, Bramley J. Murton

**Affiliations:** 1 NGU, The Geological Survey of Norway, Trondheim, Norway; 2 Universite de Paris, Institut de physique du globe de Paris, CNRS, Paris, France; 3 GEOMAR Helmholtz Centre for Ocean Research, Kiel, Germany; 4 NOC, Southampton, United Kingdom

**Keywords:** Tectonics, Palaeomagnetism, Structural geology

## Abstract

Spreading processes associated with slow-spreading ridges are a complex interplay of volcanic accretion and tectonic dismemberment of the oceanic crust, resulting in an irregular seafloor morphology made up of blocks created by episodes of intense volcanic activity or tectonic deformation. These blocks undergo highly variable evolution, such as tilts or dissection by renewed tectonic extension, depending on their positions with respect to the spreading axis, core complexes, detachment or transform faults. Here, we use near-seafloor magnetic and bathymetric data and seismic profiles collected over the TAG Segment of the Mid-Atlantic Ridge to constrain the tectonic evolution of these blocks. Our study reveals that the presence and evolution of oceanic core complexes play a key role in triggering block movements. The deep subvertical detachment fault roots on the plate boundary, marked by a thermal anomaly and transient magma bodies. Thermal and magmatic variations control the structure and morphology of the seafloor above the subhorizontal detachment surface, occasionally leading to relocating the detachment.

## Introduction

Unlike fast-spreading centers associated with intense volcanic activity, slow-spreading ridges are characterized by lower magma flux^1^ and tectonic extension, often accommodated by long-lived detachments faults^2–5^. As a result, slow-spreading seafloor exposes variably faulted and tilted crustal blocks, dykes, lower oceanic crust and mantle rocks, comprising oceanic core complexes (OCCs)^6,7^. Due to its importance as a host to massive sulfide deposits, the slow-spreading TAG Segment (26°N, Mid-Atlantic Ridge, spreading rate 23.2 mm/y) was surveyed in 2016 during cruise M127 of German R/V *Meteor* by the Autonomous Underwater Vehicle (AUV) *Abyss* to collect high-resolution, near-seafloor magnetic and bathymetric data. Seismic reflection data were also acquired using airgun shots and a deep-towed multichannel seismic streamer. Hydrothermal massive sulfide mounds usually form prominent bathymetric features associated with a strong magnetic signature reflecting the geological setting, and are therefore easy to identify^8–15^. The magnetic signature of these hydrothermal sites is not always centered above the features. This mismatch is either interpreted as a consequence of the tectonic tilt of a homogeneous underlying block^16^ or of a composite, heterogeneous geology of the basement. An additional parameter to be considered is the possible inclination of the hydrothermal conduits. We combine the magnetic response of hydrothermal mounds at different altitudes above the seafloor with high-resolution bathymetric data to constrain the geology, tilt and conduit inclination, unveiling the detailed tectonics of the TAG segment (See “Methods”).

## Generalities

When the basaltic oceanic crust cools, its magnetic minerals acquire a remanent magnetization aligned with the ambient geomagnetic field. The resulting magnetic anomaly is generally made of two lobes and is difficult to interpret. To alleviate this difficulty, the anomaly is reduced to the pole (RTP), where both the magnetization and geomagnetic field vectors are vertical and the anomaly located above its causative body. Nevertheless, subsequent tectonic events may tilt crustal blocks, affecting the magnetization vector orientation and therefore the shape of magnetic anomalies^16^.

At basalt-hosted hydrothermal systems, high-temperature fluids demagnetize basalt permanently due to the alteration of titanomagnetite^17–20^. Assuming the crustal block hosting the hydrothermal mound is geologically homogeneous and has not been tilted, the RTP magnetic anomaly is a low centered over the bathymetric features. For hydrothermal mounds growing on a homogeneous and tilted basaltic block, the RTP magnetic anomaly is no longer a magnetic low. The correction required to transform this anomaly to a magnetic low provides an indirect way to approach the underlying block magnetization vector direction. The orientation of hydrothermal conduits (for instance along faults) is a possible cause of apparent tilt (Supplementary Information). Analyzing magnetic surveys acquired at different altitudes allows discriminating between these two effects.

Conversely, ultramafic-hosted hydrothermalism produces magnetization^21^ aligned with the geomagnetic field due to the formation of abundant magnetite. In this case, any tilt unraveled by the magnetic anomaly analysis happened subsequently to the hydrothermal mound formation and is therefore very recent.

**Figure 1:**
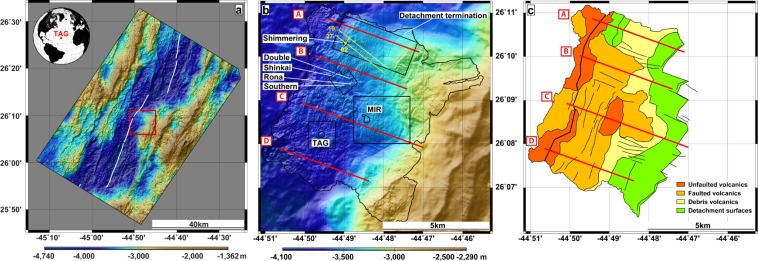
Bathymetry and interpreted morphology of the study area. (**a**) Regional, ship-based bathymetry of the TAG Segment (Mid-Atlantic Ridge 26°N). Red box delineates **b**. White lines mark the spreading axis. (**b**) High-resolution bathymetry acquired by AUV *Abyss* overlay on regional bathymetry. Black boxes delineate Supplementary figures. Red lines mark the location of schematic cross-sections A–D shown in Fig. 3, yellow lines indicate the location of seismic profiles shown in Fig. 4. Annotations point out known hydrothermal sites. (**c**) Seafloor morphology deduced from bathymetry and direct observation. Thick black line corresponds to the spreading axis, thin black lines to major faults and other tectonic elements.

Active and inactive massive sulfide deposits on the TAG Segment were surveyed by AUV *Abyss* at altitudes ranging from 20 to 120 m (Fig. 1, Supplementary Material). In the northern part of the survey area, these data are complemented by multichannel seismic reflection profiles imaging detachment surfaces. Combined with previously published data from the TAG Mound^8^, our new datasets represent a unique opportunity to constrain the tectonic history of the segment.

## Observations

**Figure 2:**
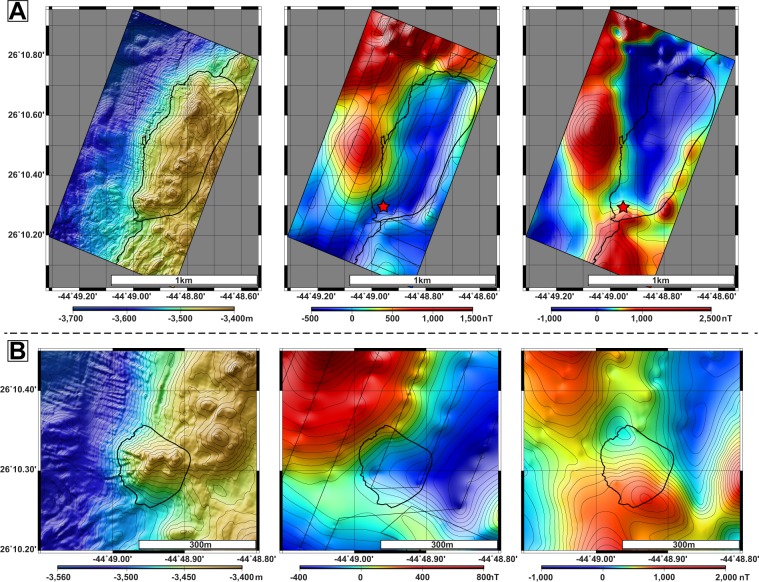
Bathymetry and magnetic anomaly of Shimmering. (**A**) High-resolution bathymetry, non-RTP magnetic anomaly (with AUV routes) and RTP magnetic anomaly over Shimmering Hill. (**B**) High-resolution bathymetry, non-RTP magnetic anomaly (with AUV routes) and RTP magnetic anomaly over hydrothermal site Shimmering.

A recent study based on deep-sea submersible magnetic data collected ~20 m over active basalt-hosted hydrothermal site TAG revealed that the associated RTP magnetic anomaly low^8–13^ remains dipolar with a minimum located south of the hydrothermal mound^16^. Correcting the RTP anomaly requires a ~50° block tilt with a rotation axis parallel to the spreading axis, i.e., the tilt occurs in a N120°E direction (counted positive if an initially horizontal surface dips toward this direction) assuming a vertical hydrothermal conduit^16^ (Supplementary Information). The resulting low is located above the site. AUV *Abyss* acquired magnetic data ∼70 m above the TAG Mound. The RTP magnetic anomaly low associated with TAG is located ∼100 m northwest of the mound on this dataset (Fig. 2). The shift between magnetic lows at ~20 m and ~70 m reflects a ~60° inclined hydrothermal conduit trending N60°W. Such inclination in turn slightly distorts the anomaly, accounting for 12° of the estimated tilt (Supplementary Information). The real block tilt is therefore ~38° towards a 120° direction. The 38° block tilt adequately corrects the magnetic signature of neighboring volcanic mounds on the AUV data (Supplementary Information). The uncertainty on this value is very low (~5°) because magnetic data from the *Alvin* were collected close to the seafloor (~20 m above the highest point of the hydrothermal mound) and the prominence of hydrothermal site TAG makes replacing the magnetic anomaly above its causative source straightforward.

Zone Mir^22^, a massive sulfide mound 2 km ENE of the TAG Mound (Fig. 1), was surveyed by AUV dives at different altitudes. Unlike TAG, the RTP magnetic anomaly does not require correction to display a magnetic low, implying the underlying block is not significantly tilted. The magnetic low shifts westwards as the AUV altitude increases: as for the TAG Mound, data acquired at different altitudes unveil a 60° inclined hydrothermal conduit trending N60°W (Supplementary Information).

Several inactive hydrothermal mounds lie within a ~200 m deep depression ~2 km NNW of Mir (Fig. 1). Magnetic datasets were collected at three altitudes over these mounds. On the highest resolution data (closest to the seafloor), each hydrothermal mound is associated with a distinct negative magnetic anomaly (Supplementary Information), whereas a volcanic edifice nearby displays a typical positive magnetic anomaly. At all AUV altitudes, each anomaly is centered on its source, suggesting no block tilt and a vertical hydrothermal conduit for all sulfide mounds.

A fourth area, 4 km NNE of the TAG Mound, investigated the Shimmering Mound with an AUV dive at 70 m altitude (Fig. 1 and Supplementary Information). This active hydrothermal mound protrudes above a steep westward slope. Nearby unfaulted volcanoes indicate that the spreading axis is located at the bottom of a 1 km-long, NNE elongated bathymetric high, hereafter named Shimmering Hill^23^. We consider the Shimmering Hill and the Shimmering hydrothermal site separately, as hydrothermalism at site Shimmering has likely affected its magnetic properties.

The RTP magnetic anomaly reveals a major positive anomaly Northwest of Shimmering Hill, corresponding to the Neo-Volcanic Zone (NVZ) (Figs 1c and 2A). Such anomaly most likely results from the fresh and highly magnetized basalt constituting the seafloor in this area. By contrast, the Shimmering Hill exhibits a negative magnetic response. This apparent negative anomaly results from a sharp magnetization contrast between the highly-magnetized basalt downslope and the comparatively less magnetized material of the Hill.

In a first approach, we assume the Hill is made of homogeneous material and try to adjust the magnetization vector inclination to get a magnetic anomaly above its causative source, either positive or negative. Given the orientation and location of the Hill, the most likely tilt orientation is perpendicular to the spreading axis. Nevertheless, to get a positive magnetic anomaly over the Hill, a −50° magnetization vector inclination is required, resulting in a 130° tilt in a N120°E direction (Supplementary Information). To get a negative magnetic anomaly, a ~110° is required, resulting in a back tilt of −37° in a N120°E direction (Supplementary Information). Such tilts are geologically unrealistic, i.e., the magnetic observations cannot be explained by the tilt of a homogeneous block. The Shimmering Hill is therefore made of a heterogeneous basement. The Hill may have undergone a tectonic tilt, which amount remains impossible to estimate.

Over site Shimmering, the RTP anomaly displays a negative lobe shifted North of the site (Fig. 2B). Hydrothermal sites are either associated with a negative (basalt-hosted) or positive (ultramafic-hosted) magnetic anomalies. Here again, we try to estimate the required tilt to ensure a consistent magnetic signature over the site. The position of site at the southwestern end the Hill, allows tilting in various directions. Using these additional degrees of freedom, we systematically explored all possible tilts along all possible orientations and found that no realistic tilt can be obtained (Supplementary Information). These unrealistic tilt values rule out the possibility of a site overlying a homogeneous basement. Site Shimmering therefore lies on a complex basement, similar to that of the Shimmering Hill.

Such a complex basement is corroborated by the recovery of greenstone - microgabbro and dolerites - at the top of Shimmering Hill and suggests that Shimmering Hill is mostly made of deep crust/uppermost mantle rocks. From its smooth morphology and composite petrology, we propose that the western slope of Shimmering Hill is the detachment surface of a recently initiated OCC, the top of the Shimmering Hill being the breakaway.

**Figure 3:**
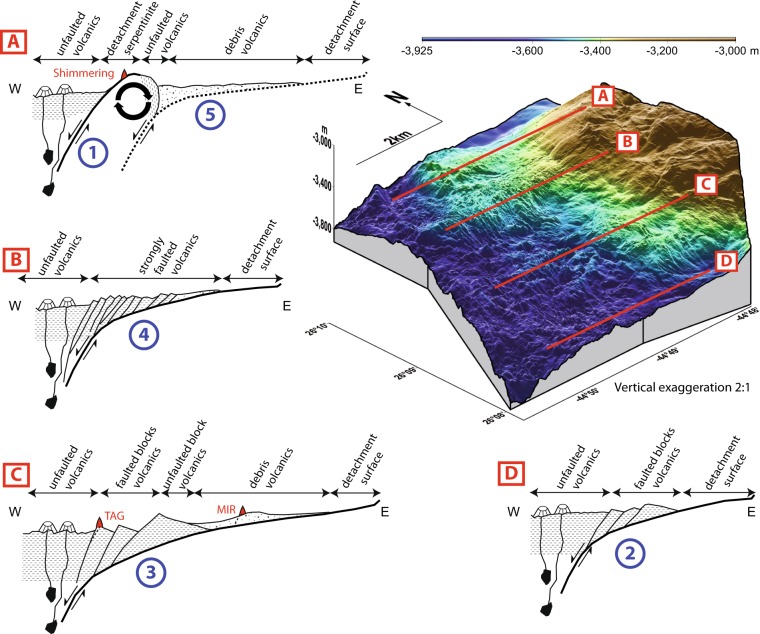
Schematic cross sections A–D and their location on a 3D bathymetric view of the study area. On the sections, thick black lines mark the detachment faults and surfaces (dashed: abandoned); thin black lines, other faults. On the left, at the ridge axis, thin black lines symbolize dykes rising from transient magma bodies to volcanoes. Red triangles represent known hydrothermal systems projected on the section. Patterns indicate volcanics (horizontal dotted lines) and debris volcanics (random points).

**Figure 4:**
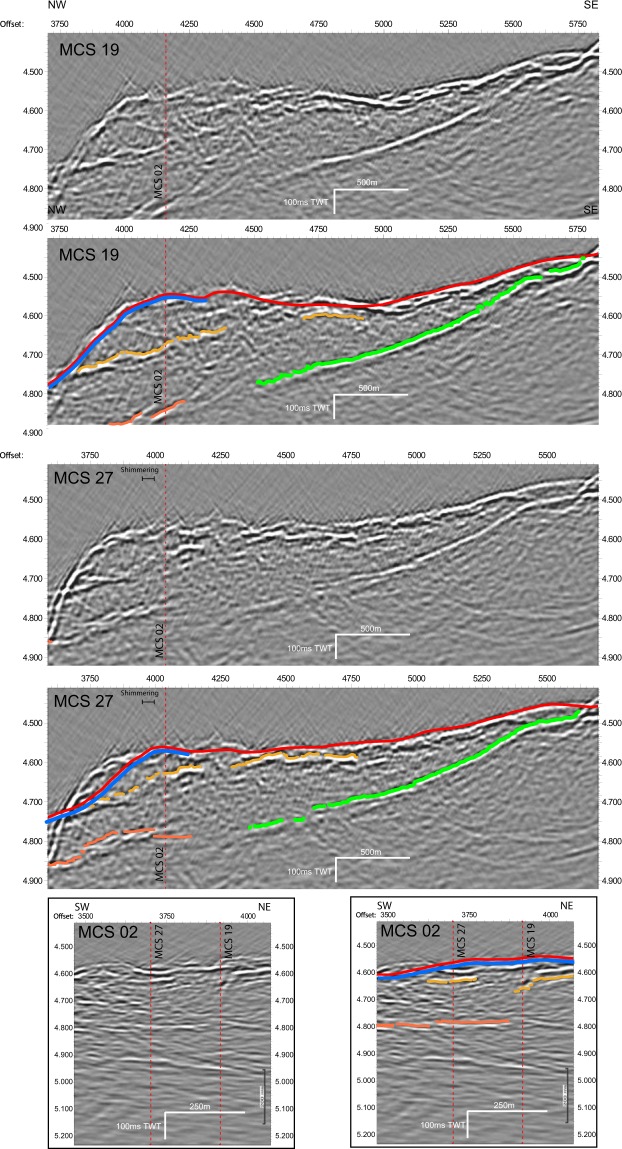
Seismic sections MCS19, 27 and 02 (see Fig. 1 for location) with interpretation of major reflections. Red vertical dashed lines indicate cross points of the seismic network. Regional bathymetry (red) is overlain on the seismic section. Discrepancies indicate the complex tectonic structure imaged in seismic data by strong side echoes. Green lines indicate the outline of the smooth dipping old detachment, blue lines mimicking the seafloor mark the steeper new detachment surface.

An older OCC has been recognized ~3 km ESE, where another NNE bathymetric high, the breakaway, overlooks a smooth, 700 m-long and 1500 m-wide outcropping, corrugated detachment surface (Figs 1 and 3). This detachment roots westward under a unit showing no volcanic construction but a finely grained surface characterized by a lack of relief, minor faults trending either NNE or ESE. The corrugations observed on the detachment surface extend below this unit and control the ESE faults. This unit is likely an accumulation of rubble dragged away by the shallow underlying detachment. This explains the weak long-wavelength magnetic anomaly corresponding to this unit (Supplementary Information), as the rubble probably exhibits a random orientation and a negligible bulk magnetization. Seismic profiles (Fig. 4) image a continuous detachment surface up to the bathymetric high of the new detachment breakaway where the event terminates abruptly.

## Tectonic Implications

We investigate the tectonics of the TAG area using AUV bathymetry and block tilts derived from magnetic data. We built four sections, labeled A-D, from the observed morphologies, tectonic elements (faults, detachments), and block tilts at or near hydrothermal sites. We distinguish four terrains based on their morphology (Fig. 1): unfaulted volcanics, faulted volcanics, debris volcanics, and detachment surfaces.

The ridge axis and a coherent tilted block located between hydrothermal sites TAG and Mir are characterized by unfaulted volcanics. The area next to the axis displays faulted volcanics in the center and South (Fig. 3, sections A, B, C and D) and a new detachment surface near Shimmering Mound. Eastwards, debris volcanics characterized by smooth morphology and fine-grained texture are prominent in the center and North (sections A, B, and C). Further east, outcropping detachment surfaces display the typical corrugations over a gently sloping detachment surface.

Detachment surface depth (Figs 3 and 4) is poorly constrained under the debris and faulted volcanics. Seismic profiling in the Shimmering area provides a depth of ~350 m beneath the debris volcanics. We anticipate that the detachment surface is deeper under faulted volcanics, with a depth of ~600 m under TAG^24–28^. Indeed, Section C successively displays the different morphologies as the detachment deepens toward the axis.

Two parameters account for the different fracturing of the volcanic material. The first one is the thickness of these volcanics, with the fault interval maintaining a regular aspect ratio in the resulting blocks. A deep detachment generates large coherent blocks bound by a few major faults, whereas a shallower one results in a dense network of small faults. The second parameter is the age of these volcanics, with faulted volcanics near the axis and debris volcanics eastwards at the foot of the detachment surface. The increasing fracturing of the volcanics with time suggests that earthquakes generated on the deep detachment fault at the axis affect the material overlying the detachment surface. Corresponding tremor has progressively broken large blocks apart, later developing a network of smaller faults and ultimately incoherent debris volcanics. In this framework, the various sections may represent different steps of a detachment evolution, from its birth to its demise (Fig. 3). These two parameters concur in shaping the morphology of the study area.

The major characteristic of a detachment is the asymmetric accretion of new lithosphere at the ridge axis. The location of the deep, subvertical detachment fault with respect to the hanging wall plate does not change as the footwall plate is progressively exhumed. The subhorizontal detachment surface affects only the shallow crust. With the presence of water, hard peridotite alters to soft serpentinite. As the footwall rises, serpentinite deforms and sags, resulting in the flattening detachment surface that carries away volcanic material formed at the axis. The amount of volcanic material determines how far from the axis the detachment surface outcrops.

Gentle slopes and corrugations consistently observed to the East of the study area support the presence of a single detachment in the whole study area. Conversely, the western part displays contrasted morphologies leading us to distinguish four corridors (Sections A-D) separated by deeper, apparently stable (as suggested by the absence of tilt) narrow transition zones. As previously suggested, variations in the thickness and age of the volcanics accreted to the detachment surface, and therefore the amount of magma produced at the axis, explain these contrasted morphologies. Ultimately, the magmatic activity is controlled by the thermal influx at the axis.

**Figure 5:**
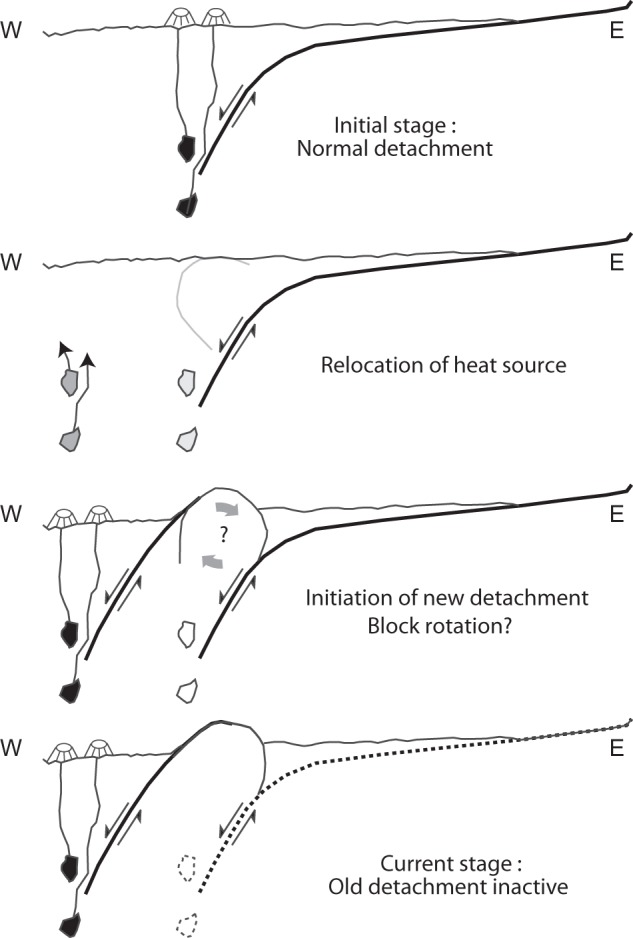
Four stages of evolution of the Shimmering Hill area illustrating the demise of the old detachment and initiation of the new one as a result of magmatic sources relocation.

The subvertical detachment fault roots on the plate boundary, at the lithosphere-asthenosphere boundary, where the thermal flux is maximal and transient magma bodies may be present. Due to the asymmetry, both the thermal anomaly and the detachment remain fixed with respect to the hanging wall plate (Sections B-D)^29^. On Section A, the magmatic activity has weakened, as testified by the thin debris volcanics and the absence of other volcanics. Moreover, the area of axial unfaulted volcanics is narrow, suggesting volcanic activity has recently resumed. In the absence of a strong thermal anomaly, bulk extension became prominent, subvertical conjugate faults (to be later rotated to the observed subhorizontal reflections) developed on the hanging wall, and the deep detachment fault was progressively dragged eastwards (Fig. 5). When the thermal anomaly and volcanic activity resumed, the old detachment was no longer properly located and ceased, and the new detachment rooted on the thermal anomaly took over.

## Methods

### Initial data processing

For deep-sea magnetic measurements collected by AUV *Abyss*, a vector magnetometer is rigidly fixed to the frame of the vehicle. Due to the proximity of the magnetometer to the vehicle, raw data are strongly affected by its magnetic effect. This influence is estimated and removed using a method developed by Isezaki^30^ and Honsho *et al*.^31^ to resolve the crustal magnetic anomaly. A calibration pattern made up of a succession of “figures-8” and ascents/descents in the N-S and E-W directions is performed at the beginning of the dive, when the AUV is far away from both the ship and the seafloor. The data collected during calibration should amount to the local geomagnetic field predicted by the International Geomagnetic Reference Field^32^ (IGRF). Any variation from this assumption is seen as a consequence of the magnetic effect of the vehicle. Using this calibration pattern, we estimate the magnetic susceptibility tensor (9 coefficients) and the remanent magnetization vector (3 coefficients) of the AUV and remove its magnetic effect from the data^31^.

Because the geomagnetic field is generally not vertical, magnetic anomalies are often made of two lobes not centered above their sources, making them difficult to interpret. Reduction to the Pole is an operator which corrects the inclination and declination of the geomagnetic field and magnetization vectors and relocate the anomalies above their sources, as if they were observed at the geomagnetic pole.

### Constraining the source of the magnetic anomalies

The depth of the dominating sources producing magnetic anomalies varies as a function of the altitude of acquisition. Data collected at a low altitude are characterized by a high amplitude and short-wavelength content corresponding to outcropping or shallow subseafloor sources. With such data, it is possible to image small dimension structures such as hydrothermal systems. The wavelength content decreases and the dominating sources deepen with increasing altitudes. Using data collected at various altitudes above a magnetized source makes possible to constrain its geometry. Two types of AUV dives were undertaken during the M127 cruise: standard dives at altitudes of 70–120 m above the seafloor for a wide regional survey (Fig. 1) and specific dives at lower altitude, focusing on hydrothermal targets. Therefore three datasets collected at various altitudes exist for the Mir and Shinkai/Southern/Rona/New Mounds areas. On the active TAG Mound, we compare high-resolution magnetic data collected by Deep-Sea Submersible *Alvin* in 1993^8^ and the German AUV *Abyss* in 2016.

Reduced-to-the-Pole (RTP) anomalies may still show some deviation with respect to their putative source. We consider two possible causes for such deviations: either the magnetization vector causing the anomalies (i.e. the underlying block of a basalt-hosted hydrothermal site or the magnetized stockwork zone of an ultramafic-hosted site) has been tilted, or the geometry of the source (for instance a hydrothermal conduit) is inclined.

We estimate possible tilt of the magnetization vector on the dataset collected at the lowest altitude, because very shallow sources dominate and the effect of slanted magnetized bodies is low. In addition, the correlation of anomalies and bathymetric features is easier on this dataset of highest resolution. We determine the optimum tilt angle that moves the anomaly above its source^16^. The other datasets are corrected using this optimal tilt angle and the position of the magnetic low on the datasets at different altitudes allow calculating the inclination of the demagnetized hydrothermal conduit. We use forward modeling to estimate the apparent tilt induced by such inclined conduit and determine the tectonic tilt^32^.

## Supplementary information

Supplementary Information

## Data Availability

Bathymetric data products from AUV dives conducted during RV Meteor cruise M127 are available under: https://doi.pangaea.de/10.1594/PANGAEA.899415.
