# Supplementary material for: Detachment tectonics at Mid-Atlantic Ridge 26°N
Source: Sci Rep. 2019 Aug 14;9:11830. doi: 10.1038/s41598-019-47974-z (PMC6694105; doi:10.1038/s41598-019-47974-z)
Supplement: Supplementary file 1 — Supplementary Information [file 41598_2019_47974_MOESM1_ESM.pdf]

Supplementary Information

Detachment tectonics at Mid-Atlantic Ridge 26°N

Florent Szitkar1*, Jérôme Dyment2, Sven Petersen3, Jörg Bialas3, Meike Klischies3, Sebastian Graber3, Dirk Klaeschen3, Isobel Yeo4 and Bramley J. Murton4

1 NGU, The Geological Survey of Norway, Trondheim, Norway
2 Institut de Physique du Globe and CNRS, Paris, France
3 GEOMAR Helmholtz Center for Ocean Research, Kiel, Germany
4 NOC, Southampton, United Kingdom
Correspondence to Dr. Florent Szitkar, florent.szitkar@ngu.no

ABSTRACT

Spreading processes associated with slow-spreading ridges are a complex interplay of volcanic accretion and tectonic dismemberment of the oceanic crust, resulting in an irregular seafloor morphology made up of blocks created by episodes of intense volcanic activity or tectonic deformation. These blocks undergo highly variable evolution, such as tilts or dissection by renewed tectonic extension, depending on their positions with respect to the spreading axis, core complexes, detachment or transform faults. Here, we use near-seafloor magnetic and bathymetric data and seismic profiles collected over the TAG Segment of the Mid-Atlantic Ridge to constrain the tectonic evolution of these blocks. Our study reveals that the presence and evolution of oceanic core complexes play a key role in triggering block movements. The deep subvertical detachment fault roots on the plate boundary, marked by a thermal anomaly and transient magma bodies. Thermal and magmatic variations control the structure and morphology of the seafloor above the subhorizontal detachment surface, occasionally leading to relocating the detachment.

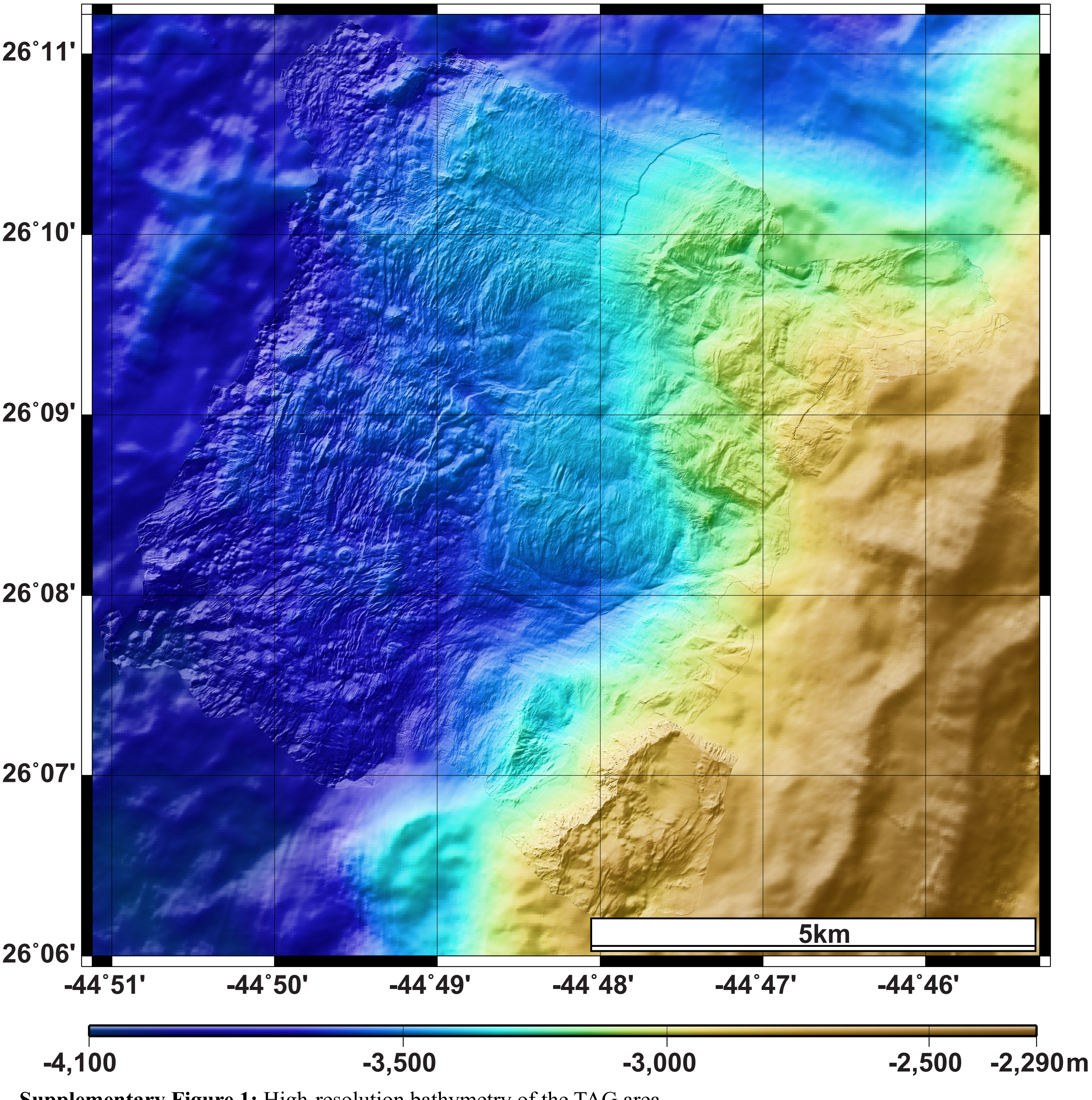

Supplementary Figure 1: High-resolution bathymetry of the TAG area

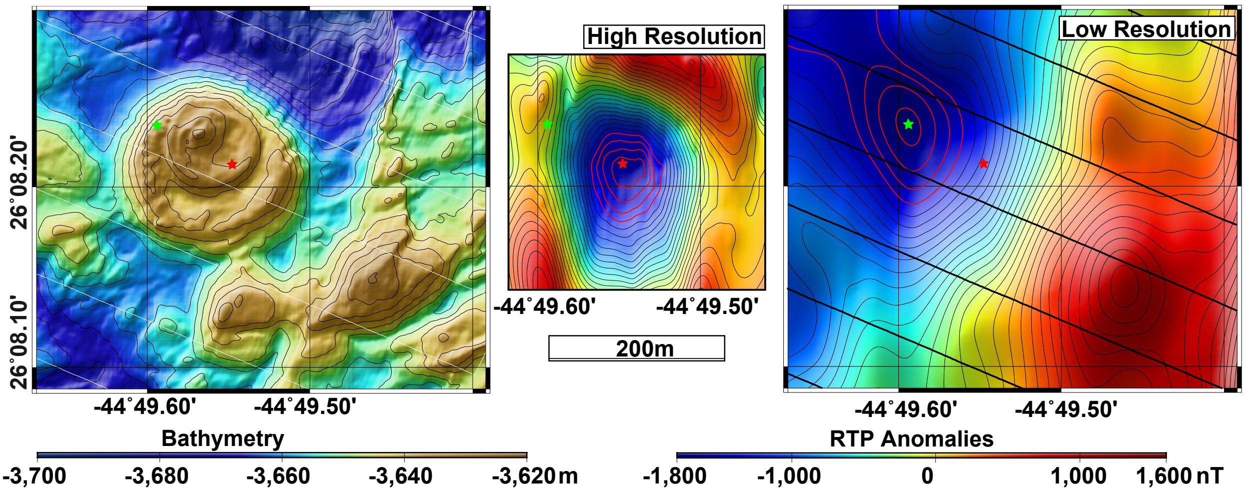

Supplementary Figure 2: Bathymetry and magnetic anomaly of the basalt-hosted hydrothermal mound TAG. (a) High-resolution, near-seafloor bathymetry. (b) Reduced-to-the-pole (RTP) magnetic anomaly at ~20 m altitude from Alvin data corrected for a 53° effective tilt. (c) RTP magnetic anomaly at ~70 m from Abyss data corrected for the same effective tilt. The magnetic low is shifted NW by 100 m with respect to the active site. A simple trigonometric calculation results in a 60° inclined hydrothermal conduit. Such an inclination generates a 12° apparent tilt, the tectonic tilt therefore amounting to 38°.

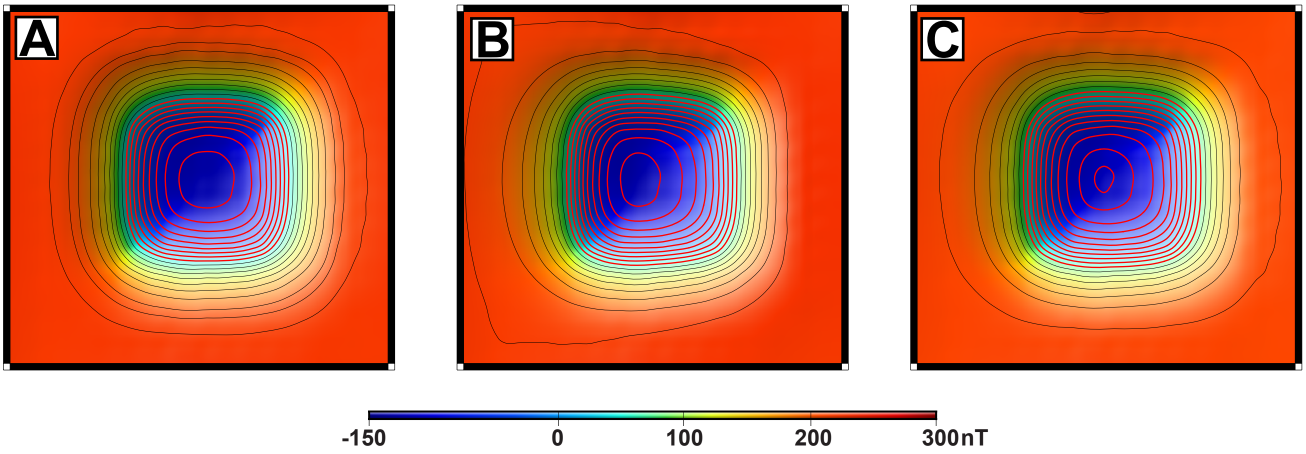

Supplementary Figure 3: Apparent tilt generated by an inclined hydrothermal conduit on near-seafloor magnetic anomaly. (a) magnetic anomaly generated by a vertical conduit at the magnetic pole (vertical magnetization and geomagnetic field vectors) at 20 m altitude. The anomaly is symmetrical. (b) idem for a 60° inclined conduit. The anomaly is slightly distorted. (c) idem for a 60° inclined conduit, corrected for a 12° Westward apparent tilt. The anomaly is symmetrical. The apparent tilt generated by an inclined hydrothermal conduit on the magnetic anomaly at 20 m is ~12°

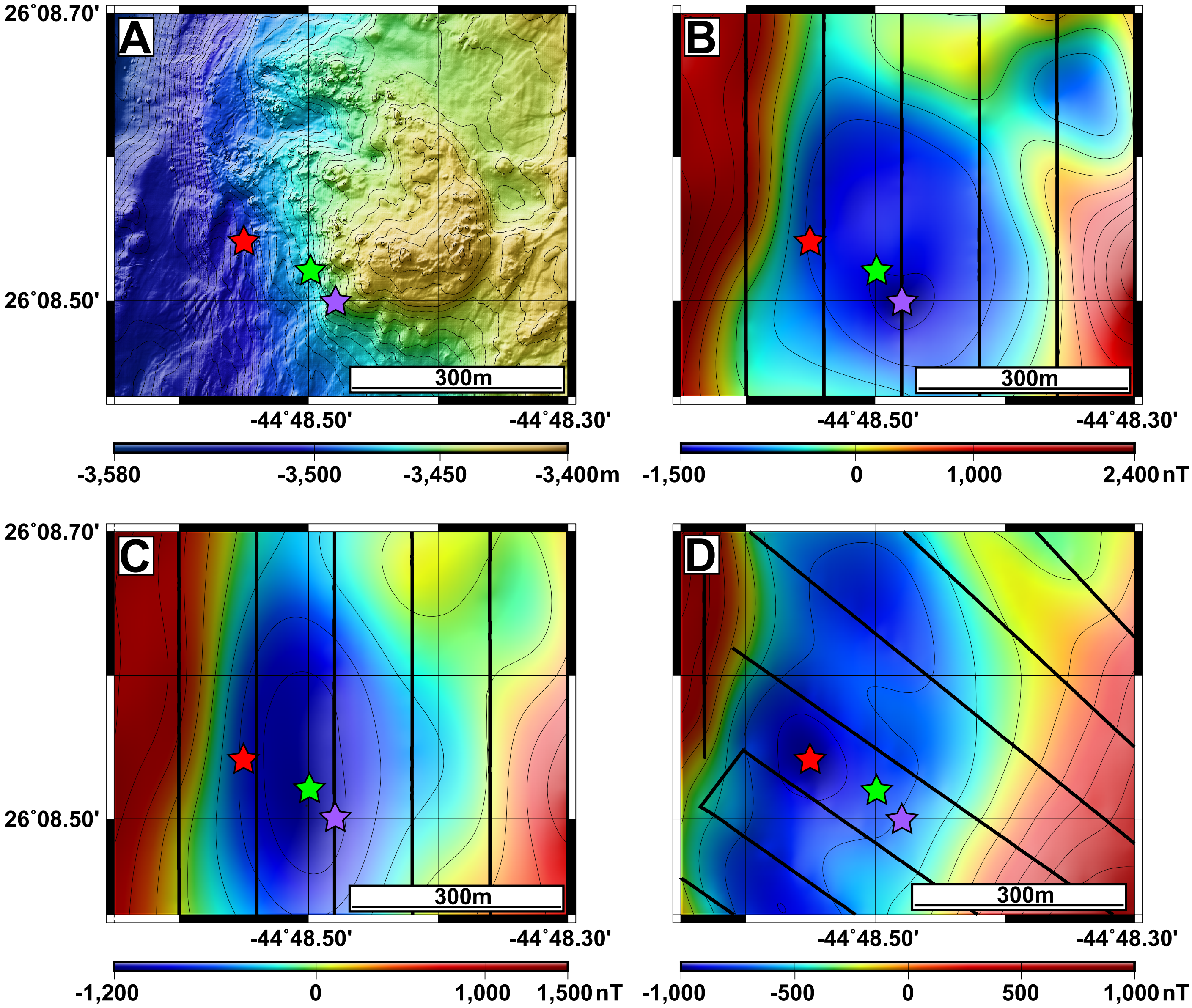

Supplementary Figure 4: Bathymetry and magnetic anomaly of the basalt-hosted hydrothermal mound Mir from Abyss data. (a) High-resolution, near-seafloor bathymetry. Reduced-to-the-pole (RTP) magnetic anomaly (b) at ~20 m altitude, (c) at ~70 m altitude, (d) at 120 m altitude. No significant tilt correction is required. The magnetic lows at different altitudes are shifted NW with respect to the active site. A simple trigonometric calculation results in a 60° inclined hydrothermal conduit. Such an inclination generates a 10° apparent tilt, which is too small to be discerned on the data.

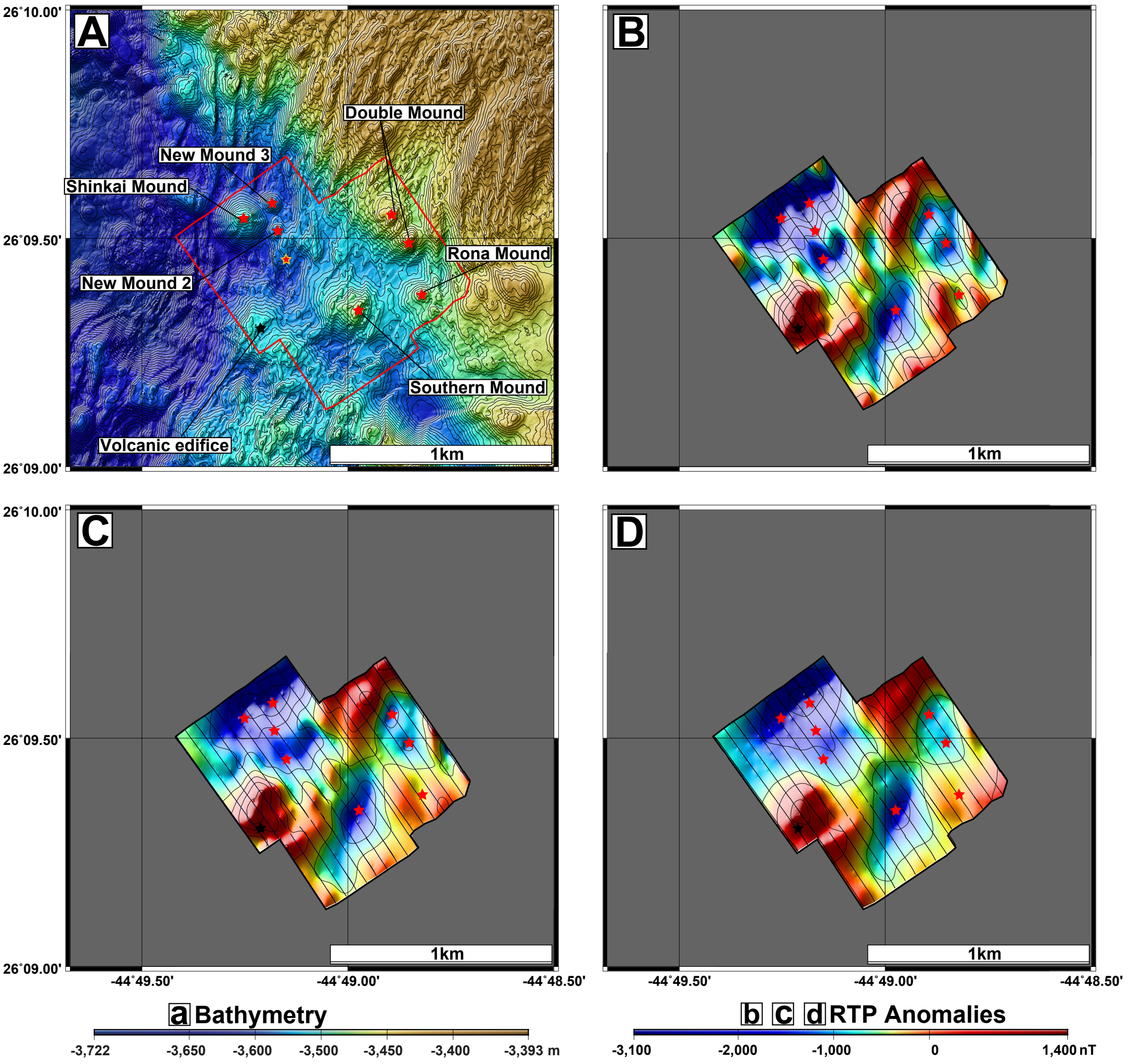

Supplementary Figure 5: Bathymetry and magnetic anomaly of the basalt-hosted hydrothermal mounds Shinkai, Southern, Double, Rona, and New 1 and 2 from Abyss data. (a) High-resolution, near-seafloor bathymetry. Reduced-to-the-pole (RTP) magnetic anomaly (b) at ~20 m altitude, (c) at ~70 m altitude, (d) at 120 m altitude. No significant tilt correction is required. The magnetic lows at different altitudes are located above their sources, suggesting vertical pipes.

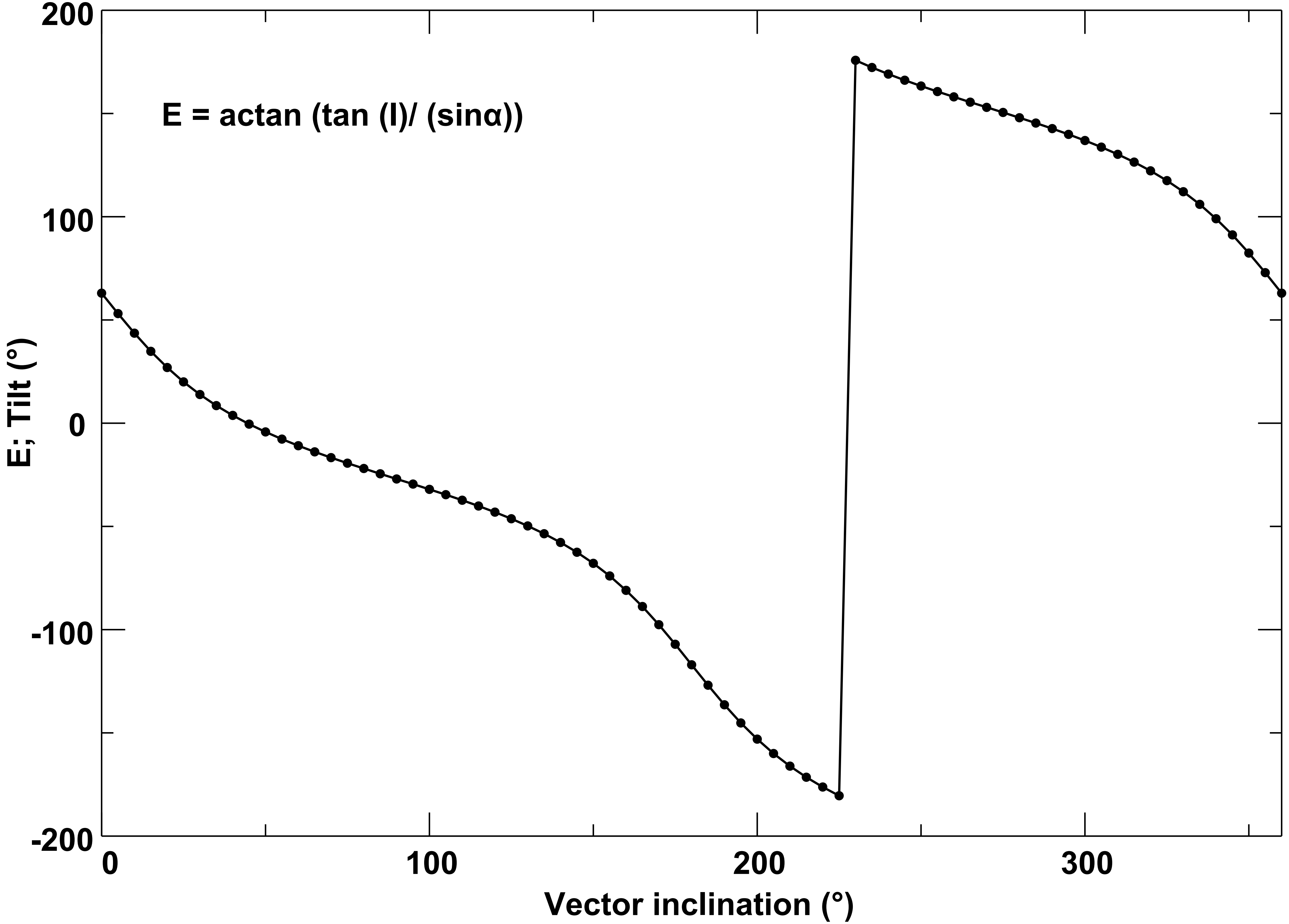

Supplementary Figure 6: Tilt as a function of the field vector inclination. The tilt, also called apparent inclination is defined as E = actan (tan (I) / sin (α)), where I is the vector inclination (counted positive downwards) and α = Azimuth - Declination. In our case, the declination is considered negligible, as the survey are is located in the immediate vicinity of the Neo-Volcanic Zone. The oceanic crust is therefore much younger than the last magnetic polarity reversal.

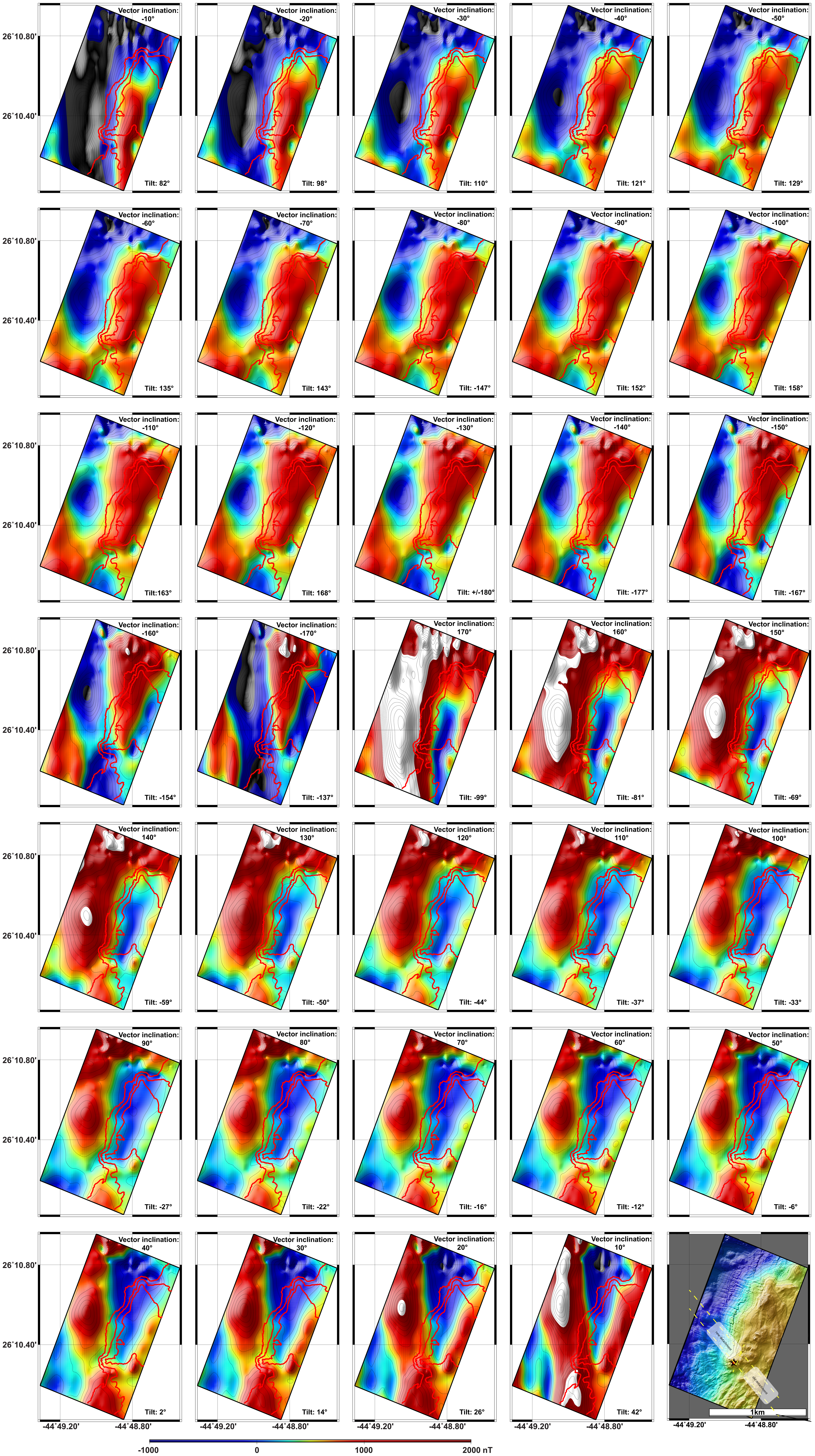

Supplementary Figure 7: Variation of the magnetic anomaly with the inclination of the magnetization vector over the Shimmering Hill. Red lines delineate the Hill based on bathymetic considerations. The corresponding tilt is estimated in a N120° direction. In order to get a homogeneous magnetic signature, the tilt must be either 130° in a N120°E (for a positive magnetic signature) direction or -37° (for a negative magnetic signature). None of these values seem realistic, ruling out the hypothesis of a geologically homogeneous hill.

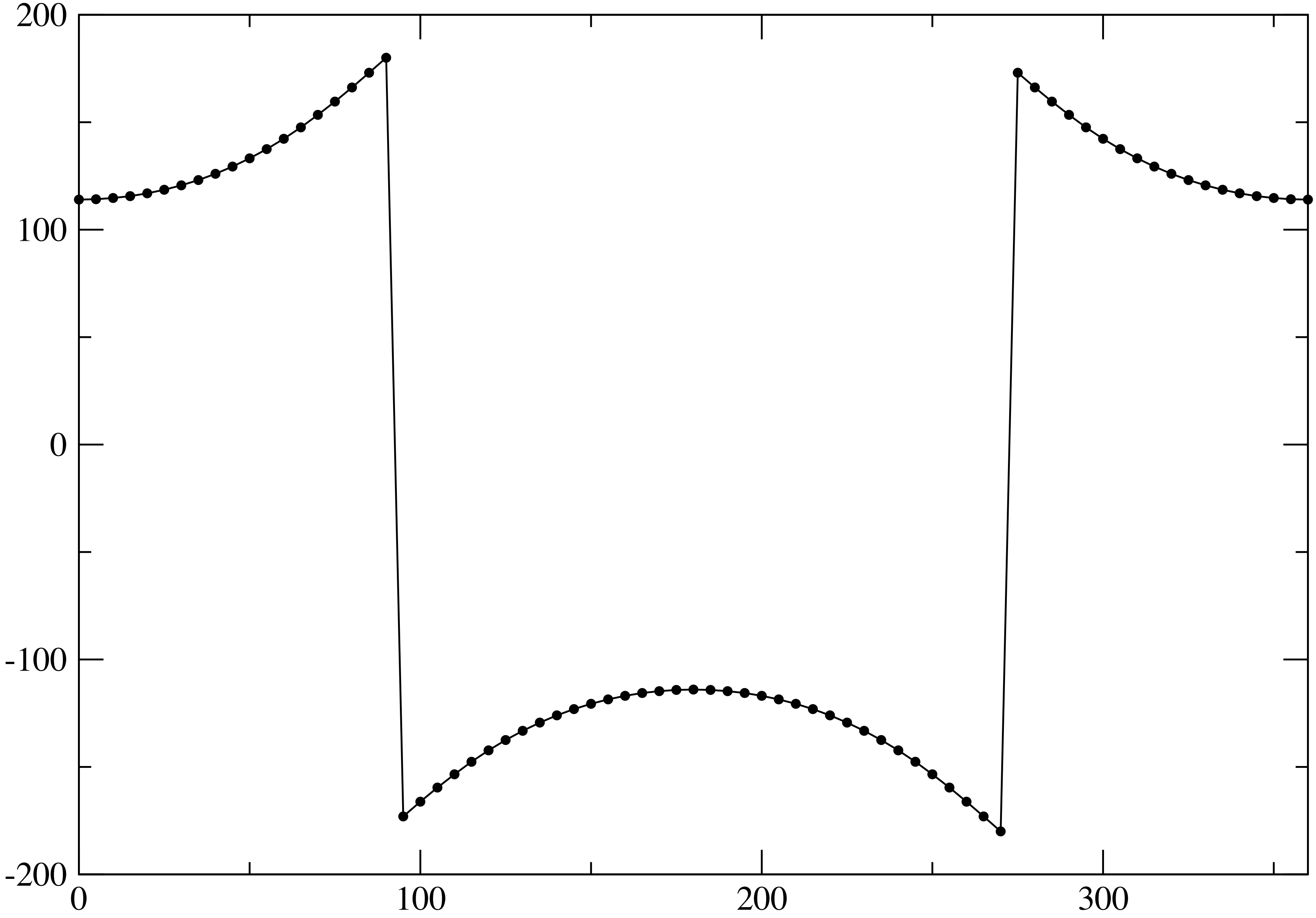

Supplementary Figure 8: Tilt as a function of the field vector declination. The tilt, is estimated every 5° assuming a 40° magnetization vector inclination. None of the values are geologically realistic, ruling out the hypothesis of a hydrothermal site sitting on top of a homogeneous basaltic or ultramafic basement.

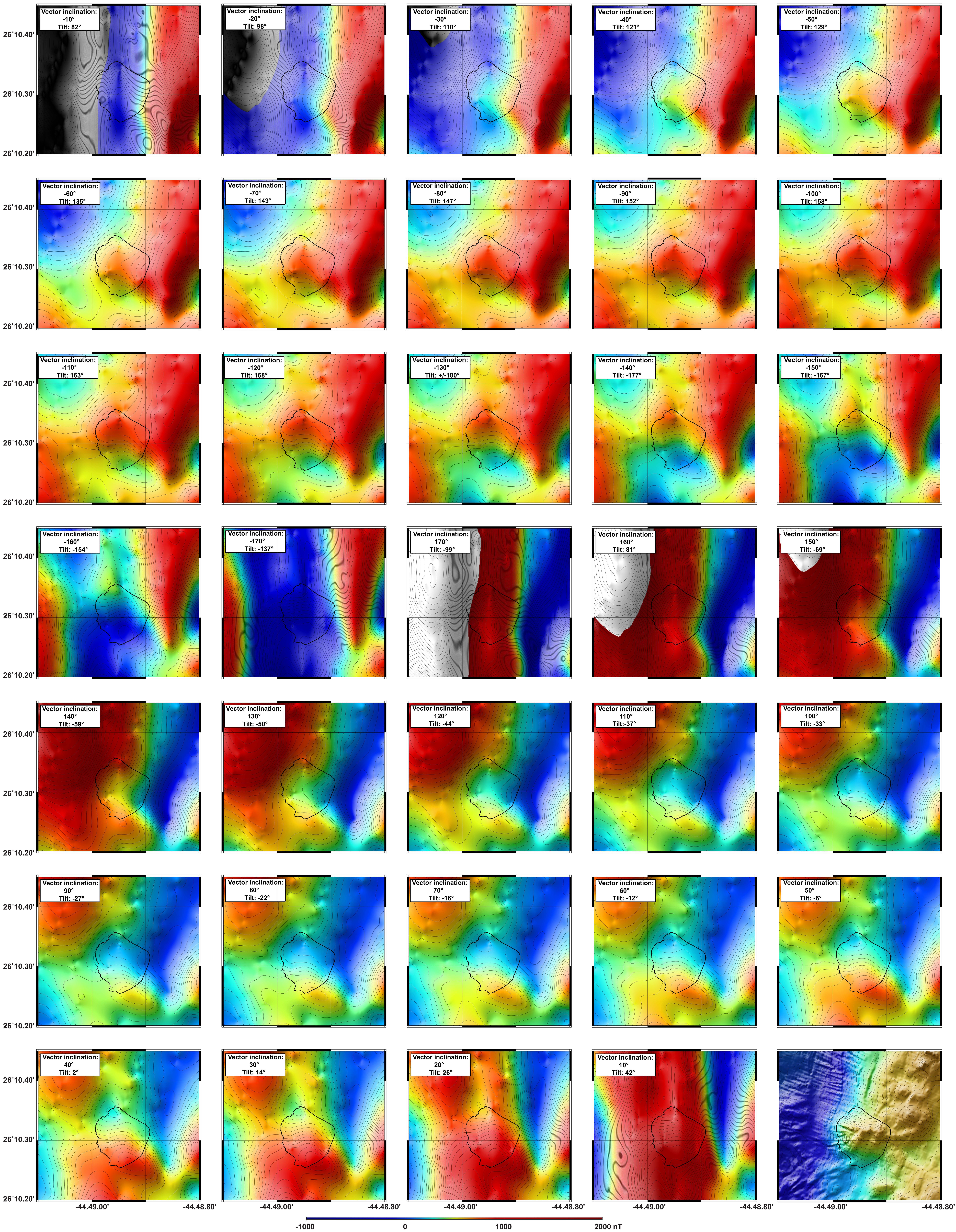

Supplementary Figure 9: Variation of the magnetic anomaly with the inclination of the magnetization vector over site Shimmering. Black lines delineate the hydrothermal mound based on bathymetic considerations. The corresponding tilt is estimated in a N120° direction. Hydrothermal sites are either associated with a negative RTP magnetic anomaly (basalt-hosted sites) or a positive RTP magnetic anomaly (ultramafic-hosted sites). In this case, the tilt or back-tilt that must be considered to get a homogeneous magnetic signature (143° in a N120°E dirction to get a positive magnetic signature and -37° to get a negative magnetic signature) are not realistic. We therefore prove that either the tilt occurs with another axis direction or the site is not associated with a clear magnetic signature.
